# *metaKEGG*: A comprehensive algorithm package to visualize multi-omics pathway enrichment

**DOI:** 10.1016/j.bbrep.2026.102723

**Published:** 2026-08-25

**Authors:** Michail Lazaratos, Neele Haacke, Jasmin Gaugel, Miriam Ulz, Tim Bleimehl, Justus Täger, Annette Schürmann, Heike Vogel

**Affiliations:** a German Center for Diabetes Research (DZD e.V.), München-Neuherberg, München, Germany; b Department of Experimental Diabetology, German Institute of Human Nutrition Potsdam-Rehbruecke, Nuthetal, Germany; c Institute of Nutritional Sciences, University of Potsdam, Nuthetal, Germany

## Abstract

*metaKEGG* is a comprehensive software package designed to streamline the visualization and integration of pathway enrichment results from multi-omics data, providing accessible and detailed insights into the molecular mechanisms driving health and disease. Unlike standard pipeline approaches, *metaKEGG* incorporates novel concepts allowing for clear, granular representation of gene-level or transcript-level expression changes. Beyond transcriptomic analysis, *metaKEGG* also supports epigenetic and regulatory metadata layers, such as methylation profiles and miRNA target annotations, offering users a versatile solution to depict complex regulatory interactions within a single pathway map. Its modular architecture provides nine analysis pipelines to suit various experimental designs, from comparing gene expression across multiple conditions to the integration of compound-based metabolomics data. Its implementation in Python ensures easy adoption and reproducibility, while a user-friendly web app allows researchers with limited bioinformatics expertise to harness *metaKEGG*'s full potential.

## Highlights

• MetaKEGG dynamically splits pathway nodes to show every mapped gene and transcript.
• Nine automated pipelines for expression, methylation and microRNA data.
• Multiple experiments and data types can be compared on one pathway map.
• Metabolite and lipid data can be mapped alongside gene-level pathway results.
• Python and web tools support user-friendly and accessible pathway analysis.

## Graphical abstract

**Figure:**
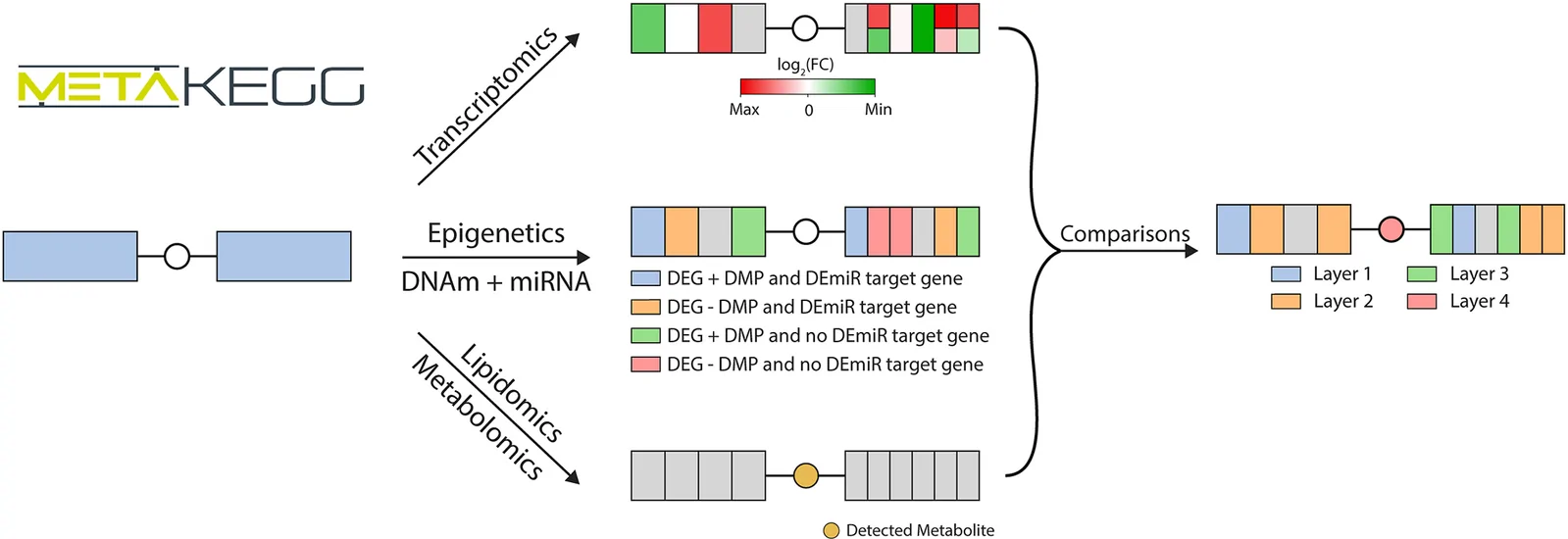

## 1 Introduction

Advances in high-throughput technologies have transformed multi-omics research by enabling comprehensive and spatially resolved single-cell profiling. Early methods such as MERFISH [1] established highly multiplexed spatial transcriptomics, while parallel single-cell sequencing approaches like scM&T-seq [2] and CITE-seq [3] linked transcriptional, epigenetic, and proteomic heterogeneity. Integrative frameworks such as Multi-Omics Factor Analysis (MOFA) enabled unsupervised analysis across modalities [4], and more recent joint profiling of chromatin accessibility and RNA has improved interpretation of regulatory dynamics and cell states [5]. These methodological advances are complemented by broader applications across drug discovery and systems pharmacology [6], spatially resolved multi-omics applied to complex diseases [7], and integrative multi-omics strategies for biomarker discovery and precision oncology [8]. Additionally, high-throughput technologies are being applied in translational research, particularly in immunology and cancer, where multi-omics is revealing new therapeutic opportunities, including natural killer cell immune checkpoints [9] and host-microbiome interactions that shape tumor biology, immune responses, and treatment outcomes [10]. Together, these insights highlight the power of multi-omics to unravel the interconnected networks in disease biology and drug discovery.

Advanced high-throughput technologies such as RNA sequencing (RNAseq) have enabled researchers to generate comprehensive transcriptomic profiles often resulting in large datasets comprising thousands of differentially expressed genes (DEGs). Interpreting the biological relevance of such extensive gene lists poses a significant challenge and necessitates systematic downstream analysis.

Functional enrichment analysis is often a crucial step and a potential endpoint of a differential expression analysis at the gene or transcript level. In modern high-throughput methods, where the downstream analysis features large lists of genes to be further evaluated on their biological significance, performing such analysis can become a challenging task [11,12]. One popular resource, among others, to perform such large-scale analysis through a wide range of functional annotation tools is DAVID [[13], [14], [15], [16]], whose tools are based on the integrated and comprehensive database of annotation information, the DAVID Knowledgebase [16].

Pathway enrichment analysis is a key feature of the DAVID Functional Annotation Tool. By providing a gene list, and specifying the organism, the DAVID Functional Annotation Tool will highlight the relevant KEGG pathways [[17], [18], [19]], Gene Ontology (GO) terms [20,21], Functional Annotations, such as “biological process” or “molecular function”, and others. KEGG, a widely utilized resource in bioinformatics and computational biology, provides manually curated pathway information. However, one important limitation of complex structures, such as KEGG pathways, is the lack of post-processing tools and integrated visualization solutions that are both intuitive and accessible to researchers.

**Fig. 1:**
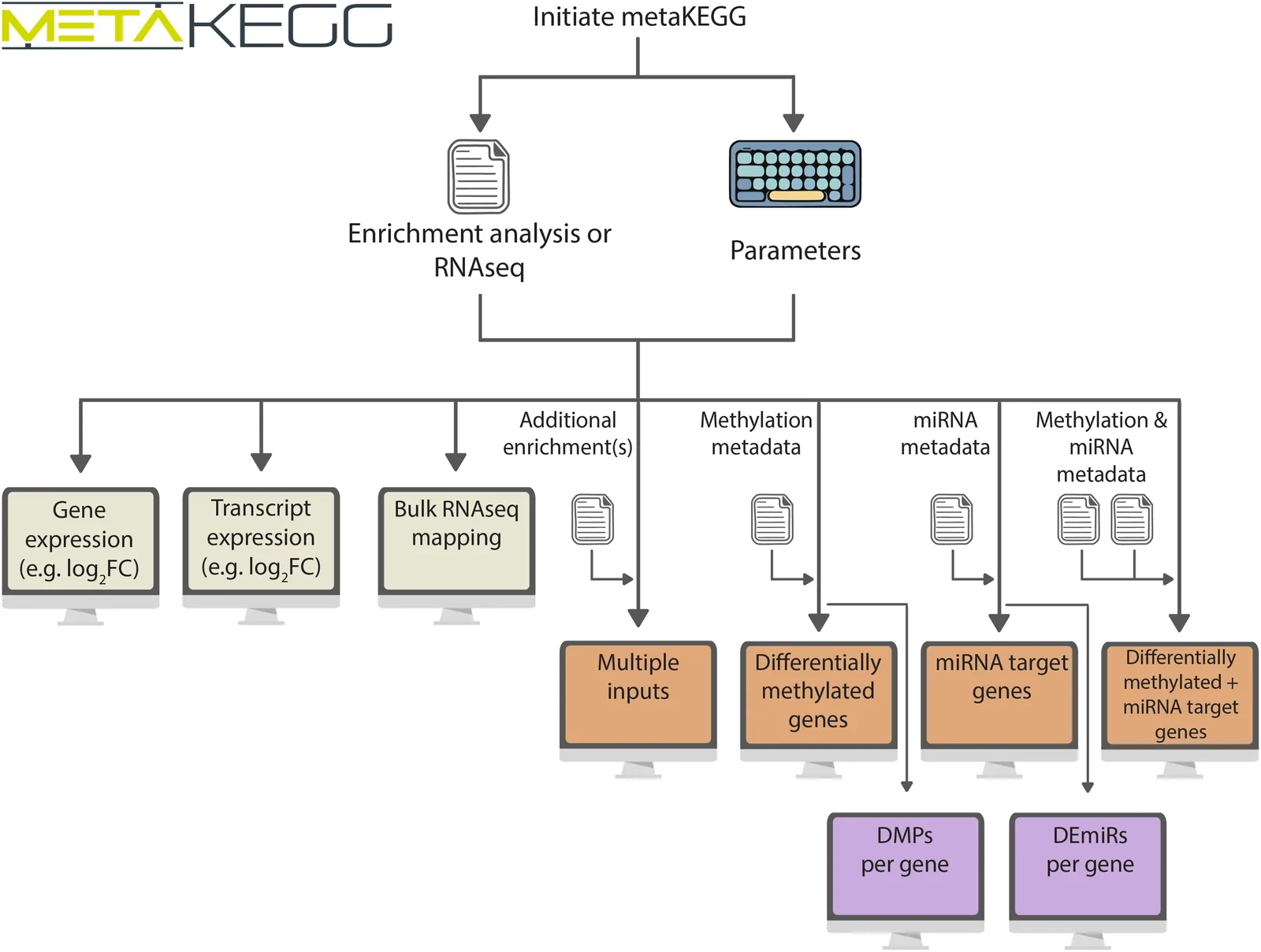
Overview of the *metaKEGG* package. Schematic representation of the pipeline structure. The user initiates the process by selecting an input file and the accompanying parameters. Next, an analysis pipeline is selected and additional parameters and input files are provided, according to the pipeline's requirements. An output folder will be generated with the analysis extension, and the corresponding analysis pipeline is executed.

To enable detailed insights into the differential regulation within individual signaling pathways, we have developed the *metaKEGG* software package (https://metakegg.apps.dzd-ev.org, https://github.com/dife-bioinformatics/metaKEGG), allowing the overlay of metadata from (multi-)omics analysis with pathway enrichment analysis outcomes. The core of *metaKEGG* is its capability to visualize differentially expressed genes/transcripts, with or without multi-layer metadata on the KEGG pathway maps. It features nine analysis pipelines (Fig. 1), namely, gene and transcript expression, bulk RNA-sequencing mapping, differentially methylated positions (DMPs) per gene, miRNA target genes, and combined methylated and miRNA target genes. *metaKEGG* uniquely integrates epigenetic data, such as DNA methylation and miRNA, and splits KEGG map nodes into sub-nodes, facilitating detailed visualization of multiple genes or transcripts per node. A notable pipeline, “Multiple inputs,” allows the comparison of gene sets affected by different interventions, conditions, or tissues, to name a few, isolating common pathways across inputs.

In contrast to existing solutions, *metaKEGG* features data-driven dynamic node splitting, in which KEGG nodes are programmatically subdivided into an adaptable number of segments, each corresponding to multiple genes or transcripts per node. The user-provided inputs enable simultaneous visualization of transcriptomic, methylation, miRNA, and/or metabolomic enrichment results. To the best of our knowledge, no existing KEGG visualization tool provides this level of flexible, data-centric, and KEGG-faithful subdivision, representing a key distinction between static overlay tools and *metaKEGG*'s dynamic visualization framework.

## 2 Results

*metaKEGG* is a fully integrated solution to bridge the gap between functional annotation analysis and visualization, using the KEGG reference pathways that most researchers are already familiar with. As the name suggests, *metaKEGG* is the first, to the best of our knowledge, that can map metadata in combination to the pathway enrichment analysis results. In addition, *metaKEGG* offers streamlined pipelines to directly visualize pathway enrichment analysis results on the KEGG reference pathways, at the gene and transcript expression level, and is capable of performing combinatorics when multiple inputs are provided.

*metaKEGG* is written in Python 3 and offers two main modes of use at the time of writing, that are intended to cover most users' needs. After installation, *metaKEGG* can be used as a library and be integrated into larger pipelines. Alternatively, analysis with *metaKEGG* can be performed through our newly launched *metaKEGG* web app (https://metakegg.apps.dzd-ev.org), hosted by the DZD (German Center for Diabetes Research) data management team. The full documentation is available in the *metaKEGG* repository. (https://github.com/dife-bioinformatics/metaKEGG). The pipelines of *metaKEGG* are fully automated after parameter definition to further benefit the potential users, resulting in a seamless and user-friendly workflow.

### 2.1 Program architecture

The *metaKEGG* algorithm package uses the results of a pathway enrichment analysis performed with DAVID (but not limited to) as input, and differential gene expression information, or other mappable information, such as *p*-values. The helper and parsing functions handle I/O, folder generation, color scales, and, most importantly, parse the input files to generate the required internal structures to perform the analyses and the API requests to retrieve the pathways’ information directly from KEGG. The drawing functions handle all graphical processes for the various analysis pipelines, including node splitting, gene mapping and generating color scales, legends and histograms. The core of the program lies within the main class which is initialized with global parameters, such as input file paths, and the analysis pipelines. The 9 individual pipelines (Fig. 1, Fig. S1) work independently from each other and perform the necessary control checks for each procedure.

### 2.2 Parsing/helper functions

*metaKEGG* parses the pathway enrichment results and gene/transcript metrics information and generates a dictionary with the pathway ID (e.g. “*mmu04932*” for the Non-alcoholic fatty liver disease pathway from *Mus musculus*) as the key, and a dictionary with additional information per pathway, such as the name and a list of genes detected in the pathway, amongst others. Using the pathway ID, *metaKEGG* employs the KEGG subpackage of Biopython [22] to perform an API request through the KEGG REST module, to retrieve, most importantly, all the gene symbols that are featured in a given pathway, and their KEGG ortholog IDs, on which *metaKEGG* relies heavily to execute its pipelines. To minimize the configuration workload from the user, we have implemented miscellaneous I/O functions to generate output folders, with the date stamp and a unique extension to the analysis types performed by the user. In contrast to existing solutions, the pipeline is executed vertically and the results are saved in the output folder generated at the beginning of the analysis. We provide a wrapping function that compiles the modified KEGG pathway with the analysis data, the original reference pathway, the color scale in a vertical and horizontal orientation or the plot legend, depending on the analysis performed.

### 2.3 Drawing functions/core

The core of *metaKEGG* lies in its drawing capabilities of differentially expressed genes/transcripts, with or without metadata on the KEGG pathway maps. After performing the annotation analysis, the user can select one of the enriched pathways, which them to the KEGG pathway map, with red star annotations on the respective nodes, for genes included in the submitted gene list, regardless of the number of genes that belong to that node. *MetaKEGG* allows users to include information on multiple gene symbols encoding the same protein, for instance, Ins1 and Ins2, each with a unique entry ID, encoding insulin.

**Fig. 2:**
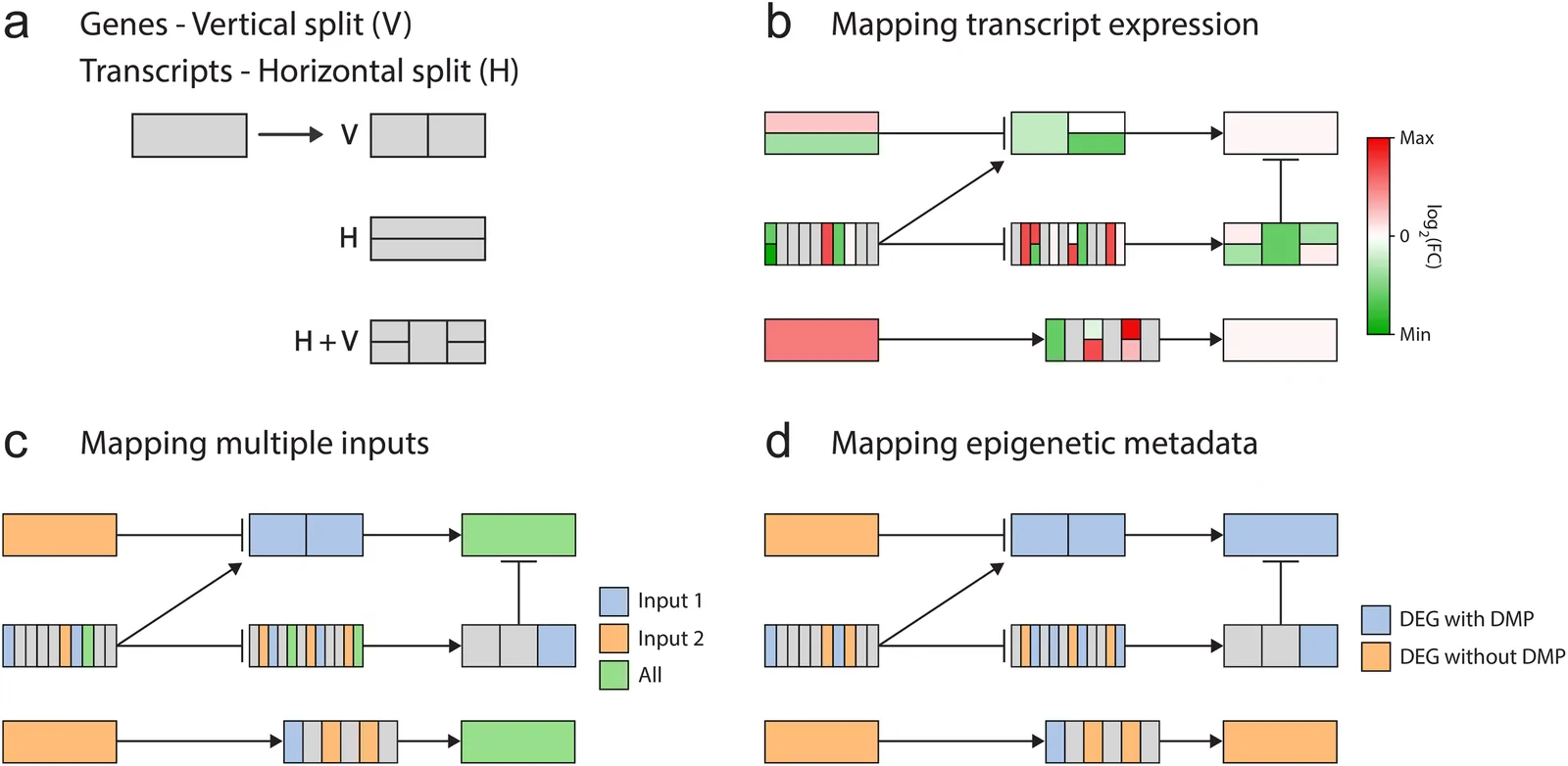
Node splitting and data mapping in *metaKEGG*. (a) Variations of node splitting. Each parent node will be split into *n* sub-nodes, corresponding to *n* associated genes. The sub-node width is dependent on *n*. Vertical splits correspond to multiple genes per parent node. Horizontal splits correspond to multiple transcripts per gene. (b) Example transcript-level mapping. Nodes are split according to the scheme shown in panel *a* and colored according to the log_2_FC values of the individual transcripts of each gene. (c) Example of dual-input mapping for a given pathway. A legend is automatically generated based on the number of input files provided, and states all possible combinations. For the case of two inputs, the categories are (input1, input2, both). Genes detected in the pathway are color-coded accordingly. (d) Example of epigenetic data mapping in *metaKEGG*. The vertical splitting protocol is applied for multiple genes per node. The additionally uploaded metadata are processed and used to generate gene-level annotations within the pathway context. DEG: differentially expressed gene; DMP: differentially methylated position.

*metaKEGG* accesses nodes from KGML-formatted files. KGML is KEGG's proprietary XML format for representing pathway elements and their relationships. For each node that contains multiple KEGG orthologs (KO), or multiple genes per ortholog, a new graphics element, or sub-node, is generated with replicated properties of the parent graphic (Fig. 2a). Then the sub-nodes are assigned a width value equal to the original node's width divided by the number of sub-nodes, and are spaced equally over the original node. In the case of transcript mapping, the process of splitting is additionally performed on each sub-node, in a vertical fashion to depict multiple transcripts per gene symbol (Fig. 2b). For a differentially expressed gene that is found in a given pathway and part of the submitted gene list, a color value is assigned according to its log_2_FC. Based on the range of log_2_FC values of the genes found in a certain pathway, a color scale bar will be automatically generated.

Based on the *Gene expression* pipeline, we have implemented an analysis pipeline that handles multiple enrichment analyses, to visualize differences and commonalities between different interventions, experiments, conditions, modules, etc. (Fig. 2c). For this instance, a list of inputs and labels are provided, and the pipeline will search and isolate common pathways that are found in all input files. The procedures for obtaining the pathway information through the API call, and the cell splitting remain the same. The pipeline will generate all possible combinations for a gene to be in. For 3 separate input files, there are 7 possible annotations for a gene, each with a unique color code. Besides “input1”, “input2”, “input3” and “all”, the pipeline will generate all pairwise combinations. Raising the input file count to 4, generates 15 possible annotations. There are 4 single inputs, 6 pair combinations, 4 triplet combinations and 1 instance for all 4 inputs.

### 2.4 Lipidomics and metabolomics

To enable the mapping of metabolites, lipids, or small compounds, we have extended *metaKEGG* to additionally accept a list of IDs. All those entities belong to the term “KEGG COMPOUND” and are distinguished by unique IDs in the format CXXXXX, where “X” is an integer, and are manually curated by the KEGG team. The user provides the list of compound IDs, and *metaKEGG* will query all pathways requested for mapping if they contain any of the provided compounds. The mapping is performed in a binary fashion, stating the presence of the compound ID, or its absence. All nine pipelines of *metaKEGG* can accept the additional compound ID list.

### 2.5 Metadata

An important aspect of *metaKEGG* is its ability to combine additional metadata on the enriched pathway (Fig. 1, Fig. 2d). The information that the pathway enrichment analysis and metadata share is the gene symbol. *metaKEGG* accepts metadata files for DNA methylation and miRNAs that include an associated gene symbol for the CpG sites, or the miRNA IDs. The two metadata categories can be mapped individually or together. Starting with a “*Gene expression*” analysis and providing one or both types of metadata, *metaKEGG* will evaluate every detected gene in an enriched pathway whether a metadata profile exists, and assign a color code that matches that profile (Table S1). In addition to binary metadata representations, *metaKEGG* offers two additional analysis pipelines that quantify the metadata in the context of the target gene. Here, the focus of the representation of the metadata changes from a binary mapping to a quantitative analysis. The methylation and miRNA implementation function in the same manner, with the exception of an additional setting for the methylation pipeline. The metadata are internally processed and organized in structures to show the number of CpG sites or the target genes of miRNAs. The metadata are grouped in bins of pre-determined ranges and are automatically adjusted according to the maximum number of metadata per gene detected for the genes of a given pathway. The genes are mapped onto a KEGG pathway and a color code is assigned for each bin, with the first bin always being dark gray, representing no available metadata for a gene, depicted as “0”, but with the genes still detected in the enriched pathway. Part of the standard output for the two quantification pipelines are two histograms for the metadata contents per pathway, one with the binned representation, and one with the distinct counts.

### 2.6 Web application of *metaKEGG*

To enhance the usability and accessibility of *metaKEGG*, we developed a web-based application (https://metakegg.apps.dzd-ev.org). The web interface (Fig. S3) offers a streamlined, stepwise workflow divided into six distinct stages, beginning with the creation of a unique URL for each analysis session and concluding with the download of compressed results. As in the programmatic usage, it has to be selected which analysis pipeline is to be performed, followed by uploading the required input file(s), and any additional metadata, depending on the selected pipeline, and finally configuring the pipeline-specific parameters. The web app will retain the results for up to 12 h. During this period, results can be downloaded or conveniently shared via the unique URL—eliminating the need to manually transfer files. Importantly, no user data is stored beyond this timeframe, and no login or registration is required, ensuring both data privacy and ease of access. The web application thus serves as an intuitive, temporary workspace designed for flexible data exchange and collaborative use. Further details about data privacy and security can be found in the “Data handling and security” section of methods.

### 2.7 Multi-omics analysis with metaKEGG

*metaKEGG* is a comprehensive software tool to enhance the visualization of pathway enrichment results and overlap metadata in terms of epigenetic layers, miRNAs, metabolomics, or lipidomics. In this section, we present example visualizations from the various pipelines available within *metaKEGG*, using randomly simulated datasets. As an illustrative case, we selected the KEGG pathway “Non-alcoholic fatty liver disease” (*mmu04932*) which is now referred to as ‘metabolic dysfunction-associated steatotic liver disease’ (MASLD), as a well-characterized and widely studied pathway. Within the aforementioned, key upstream processes associated with adipose tissue and obesity-related metabolic dysfunction are explicitly included. This reflects the pathophysiological reality that NAFLD/MASLD is not solely a liver-centric disease but rather a systemic metabolic disorder. Dysfunctional adipose tissue—characterized by increased lipolysis, altered adipokine secretion, and chronic inflammation—provides excess free fatty acids and inflammatory signals that directly contribute to liver fat accumulation [[23], [24], [25]]. By showcasing these adipose tissue-related components of the pathway, we emphasize the critical role of adipose tissue in the context of MASLD.

**Fig. 3:**
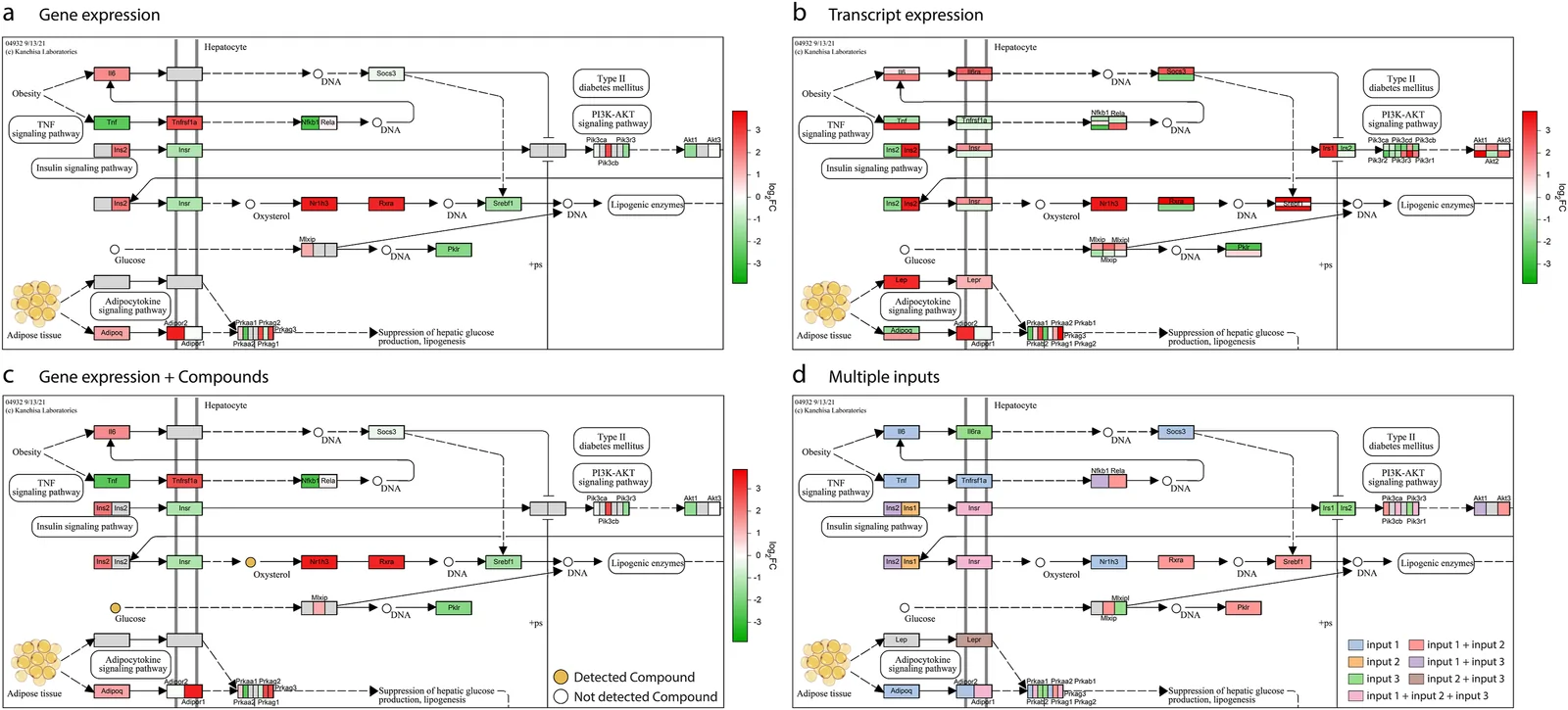
Overview of *metaKEGG* pipeline results. Panels (a) and (b) show representative outputs from a single enrichment analysis at the gene and transcript levels, respectively, while panel (c) highlights the detection and mapping of pathway-associated compounds. (d) *metaKEGG* analysis using three input files that contain the common pathway “Non-alcoholic fatty liver disease”, with all seven possible combinations shown in the legend. Panels (a-d) display a cutout of the annotated pathways, and are adapted for display purposes. A cutout illustration is not shown for the “Bulk RNAseq” analysis, as it does not differ from “Gene expression” in terms of visualization. The complete annotated pathways are available in the Supplementary Information (Figs. S4–S8).

The files used for these analyses are publicly available in the *metaKEGG*' GitHub repository (https://github.com/dife-bioinformatics/metaKEGG/tree/master/examples). Among the available pipelines, the “Gene Expression” module is likely the most broadly applicable, allowing users to map log_2_ fold-change (log2FC) values from differentially expressed gene lists onto each of the enriched pathways identified (Fig. 3a, S4). This pipeline highlights one of *metaKEGG*'s main innovations: vertical node splitting to access multiple gene symbols per KEGG node. An expansion of this algorithm is the “transcript expression pipeline”, which focuses on pathway enrichment analysis from transcript IDs and mapping all transcripts corresponding to a gene symbol (horizontal splits), in addition to the mapping of all gene symbols per KEGG node (Fig. 3b–S5). Furthermore, in cases where pathway enrichment results are not available - due to statistical limitations, for instance – *metaKEGG* still allows users to explore differentially expressed genes in the context of custom, user-defined pathway lists (Fig. S6). Compounds, as defined according to the KEGG compound database, can also be integrated across all *metaKEGG* pipelines, enabling the visualization of detected metabolites, lipids and other small molecules (Fig. 3c–S7). Beyond the single input analysis capabilities, *metaKEGG* offers a pipeline for comparative meta-analysis when multiple enrichment files are provided (Fig. 3d–S8). This feature supports direct visual comparison between experimental conditions, offering valuable insights across diverse study designs - such as different tissues, cell types, mouse models, or dietary interventions.

**Fig. 4:**
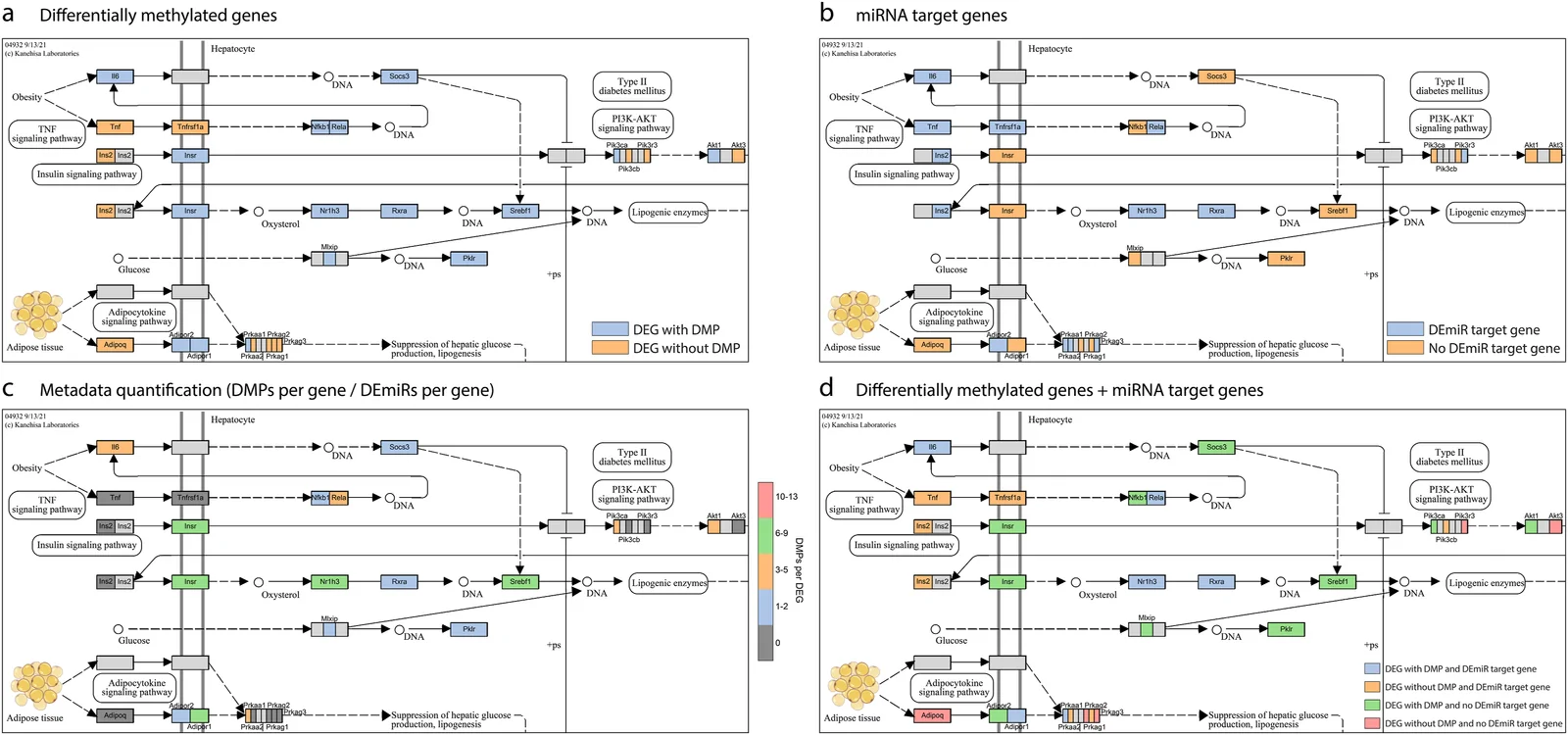
*metaKEGG* results incorporating metadata into pathway enrichment analyses. (a) *metaKEGG* analysis using methylation metadata in addition to the enrichment analysis. Genes are annotated as either “DEG with DMP” (differentially expressed genes associated with at least one differentially methylated position) or “DEG without DMP” (differentially expressed genes without associated DNA methylation changes). (b) *metaKEGG* analysis using miRNA metadata in addition to the enrichment analysis. The possible annotations are “DEmiR target gene” for a gene targeted by at least one miRNA, and “No DEmiR target gene” for a gene that is not regulated by a miRNA. (c) Quantitative representation of metadata. For simplicity, the methylation quantification is displayed using the “DMPs per gene” pipeline, reflecting the number of probes mapped to each gene. The pipeline for the quantification of the miRNA metadata (“DEmiRs per gene”) results in an equivalent visualization. (d) Combined analysis using both methylation and miRNA metadata via the “Methylated + miRNA target genes” pipeline. Each gene is assigned one of four automatically generated annotation categories, each with a distinct color code. Full pathway visualizations and associated histograms for quantitative pipelines are provided in the Supplementary Information (Figs. S9–S13).

*metaKEGG* is particularly well-suited for the integration and visualization of metadata within transcriptomics-based pathway enrichment analyses. These metadata layers may include epigenetic information (e.g., DNA methylation), miRNA target predictions, or combined datasets. In the standard configuration, *metaKEGG* offers pipelines such as “Differentially methylated genes” (Fig. 4a, S9), “miRNA target genes” (Fig. 4b–S11), and “Differentially methylated + miRNA target genes” (Fig. 4d–S13), which utilize user-provided data to generate binary annotations, as described in the Methods section. In the extended implementations, *metaKEGG* also supports quantification of the provided metadata through the “DMPs per gene” (Fig. 4c–S10) and “miRNA target genes” (Fig. 4c–S12) pipelines and transforms the binary representations of the metadata presence into quantitative ones. Quantitative metadata are additionally summarized in both grouped and ungrouped histogram formats, included as part of the standard output (Figs. S10 and S12).

### 2.8 Significance of metaKEGG

In its first iteration, *metaKEGG* was applied to interrogate lipid-gene interactions in a mouse model of type 2 diabetes (T2D), providing an integrated view of metabolic dysregulation across tissues [26]. The New Zealand Obese (NZO) mouse is a polygenic model of obesity, insulin resistance, and T2D, characterized by pronounced sexual dimorphism in diabetes susceptibility. Male NZO mice exhibit a high incidence of T2D, particularly under high-fat dietary conditions, progressing from obesity to severe insulin resistance and β-cell failure [[27], [28], [29]]. In contrast, female NZO mice, despite comparable obesity, show a lower and more heterogeneous diabetes incidence [30]. When maintained on a 60% high-fat diet, approximately 40% develop hyperglycaemia, whereas the remaining mice preserve normoglycemia. Notably, diabetes-prone (DP) and diabetes-resistant (DR) females can already be distinguished at week 10, prior to the onset of T2D [31,32]. In diabetes-prone female NZO mice, targeted lipidomics uncovered early remodelling of the liver lipidome before the onset of hyperglycaemia, marked by elevated triacylglycerols (TAGs) and reduced polyunsaturated fatty acids (PUFAs) relative to diabetes-resistant animals [27]. Remarkably, 82 of 86 altered hepatic lipid species overlapped with human plasma lipids associated with T2D or cardiovascular disease (CVD), underscoring the translational relevance of the findings.

Pathway enrichment pinpointed sphingolipid metabolism (KEGG: mmu00600) as a key affected pathway. When lipidomic data were integrated with transcriptomic profiles using *metaKEGG*, a striking tissue-specific pattern emerged: although sphingolipids were extensively altered in the liver, only five sphingolipid-related genes were differentially expressed at the transcriptional level. In contrast, gonadal white adipose tissue (gWAT) exhibited a robust response, with 31 genes-rising to 57 when sphingolipid subpathways were included-showing differential expression. By jointly mapping lipid classes and genes onto KEGG pathways, *metaKEGG* revealed an active adipose-liver sphingolipid axis and uncovered novel lipid-gene associations, including ceramide Cer(22:0), that may drive T2D risk.

A second application of *metaKEGG* focused on an exploratory analysis of the randomized crossover ChronoFast [33] trial, which enrolled 31 women with overweight or obesity [34]. The study compared two interventions, each lasting two weeks: early TRE (eTRE, 8 a.m. to 4 p.m.) and late TRE (lTRE, 1 p.m. to 9 p.m.), to assess their effects on the plasma lipidome. While no significant differences were detected between eTRE and lTRE when directly compared across lipid species, classes, or enzyme activity indices, within-intervention analyses revealed that eTRE elicited more pronounced lipidomic changes. Specifically, eTRE was associated with reductions in 103 lipid species, including ceramides and phosphatidylcholines. Using *metaKEGG*, plasma lipidomic alterations were integrated with gene expression data from subcutaneous adipose tissue (SAT) through lipid pathway enrichment and joint pathway visualization. This integrative analysis identified glycerophospholipid metabolism as a key pathway selectively affected by eTRE and highlighted three phospholipase genes, *PLB1*, *PLA2G6*, and *PLA2G4B*, whose expression differed according to the timing of food intake, linking meal timing to coordinated lipid and transcriptional regulation.

**Fig. 5:**
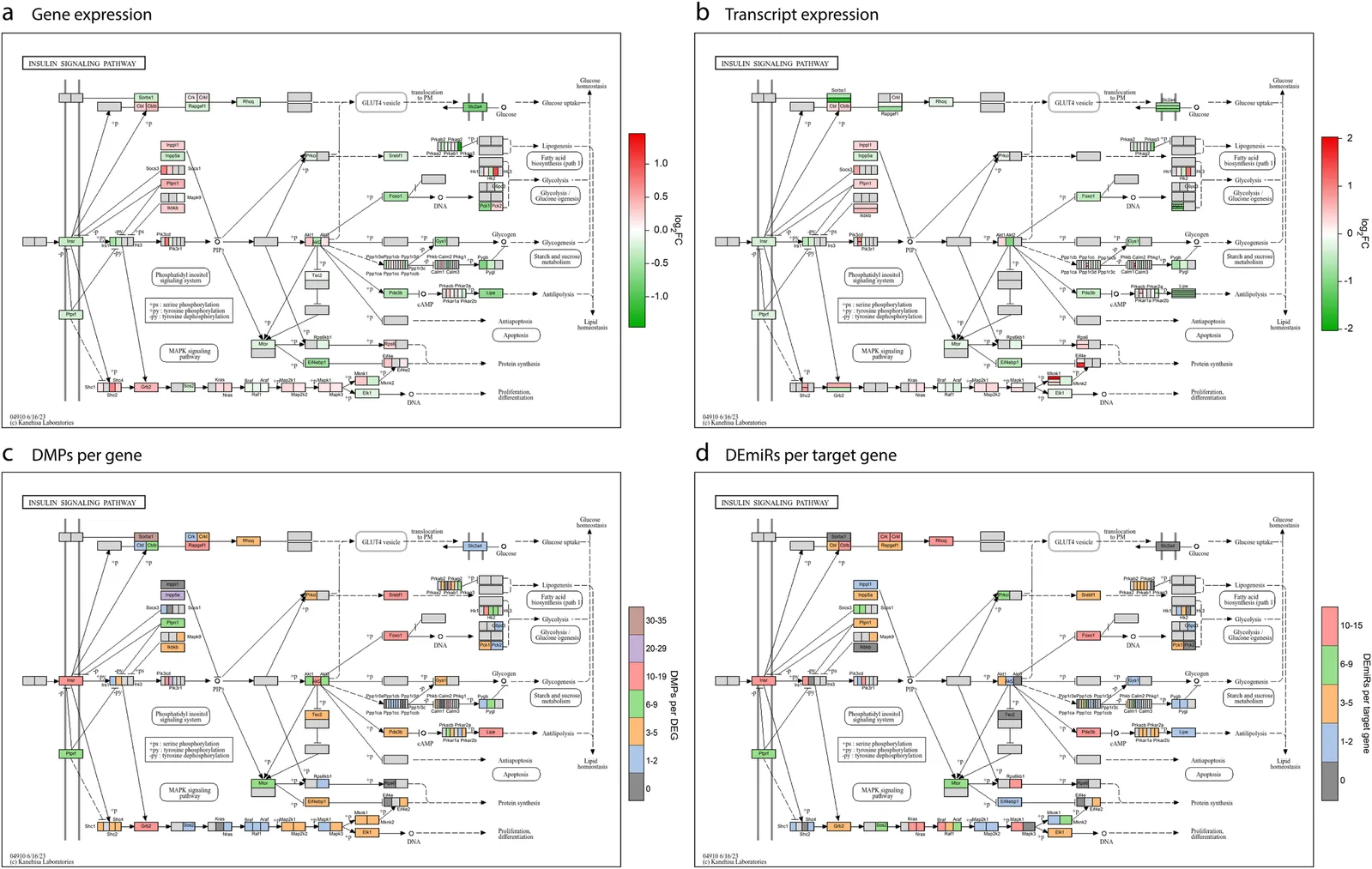
*metaKEGG* results of the NZO mouse model comparing diabetes-prone and diabetes-resistant mice, integrating transcriptome, methylome, and miRNome data. The ‘Insulin signaling pathway’ (KEGG: mmu04910) is shown across all panels. (a) Analysis of the enriched pathway at the gene level. Nodes are split vertically and genes are mapped based on their log2FC values. (b) Analysis of enriched pathway at the transcript level. Nodes are split vertically and horizontally and transcripts are mapped based on their log2FC values. (c) Quantitative integration of methylome data, showing differentially expressed (DE) genes within the pathway annotated by the number of associated methylation probes. (d) Quantitative integration of miRNA data, showing DE genes annotated by the number of differentially expressed miRNAs targeting them.

As a final application of *metaKEGG*, we performed an additional analysis on multi-omics data of gWAT from DP and DR female NZO mice [26]. Gene expression data from DP vs DR mice were processed through a standardized pipeline prior to downstream analyses. Low-abundance genes were removed using FPKM-based filtering to reduce technical noise and improve robustness. Differential expression was assessed using Mann-Whitney tests, and genes with p < 0.05 were retained for functional enrichment analysis. KEGG pathway analysis identified the insulin signalling pathway as significantly enriched (FDR = 2.8E-6), which was subsequently used as a biologically relevant case study to demonstrate *metaKEGG* and support interpretation of the mouse model. DNA methylation (p < 0.05) and miRNA expression (p < 0.05) data were integrated with the differentially expressed genes by mapping annotated genes from the MM285 mouse methylation array [35] and by linking miRNAs to experimentally validated target genes [36] using miRTarBase [37], Tarbase [38] and miRecords [39], respectively. A key feature of *metaKEGG* is the visualization of gene expression log2FC, enabling rapid pathway-level interpretation. Visualization of the insulin signalling pathway (KEGG: mmu04910) (Fig. 5a and b) revealed widespread downregulation of upstream signalling components in DP compared with DR mice, indicating attenuated pathway activation. Notably, expression of the insulin receptor (*Insr*) and insulin receptor substrates (Irs family) was reduced in DP mice. In addition, several central metabolic regulators, including *Slc2a4* (GLUT4), *Foxo1*, *Srebf1*, and *Lipe*, were downregulated. These changes are consistent with impaired glucose uptake (*Slc2a4*) [40], disrupted insulin signalling (*Foxo1*) [41], diminished lipogenic capacity (*Srebf1*) [42], and diminished lipolysis and fatty acid release (*Lipe*) [43], collectively supporting a metabolically compromised phenotype in DP mice.

Collectively, these transcriptional changes reflect a metabolic state that is generally considered as susceptible for insulin resistance and consistent with the presented phenotype. A major advantage of *metaKEGG* is the gene-level resolution within pathway nodes (Fig. 5a). For example, the IRS node contains four genes, two of which are significantly downregulated. Similarly, the AKT node comprises *Akt1-3*, with *Akt2* downregulated and *Akt1/Akt3* upregulated. To enhance interpretability, nodes can be further resolved at the transcript level (Fig. 5b). All differentially expressed transcripts of *Slc2a4* and *Lipe* are downregulated, indicating that expression changes are consistent across both gene- and transcript-level analyses. Additional mechanistic insight is gained by integrating transcriptomic profiles with epigenetic layers, including DNA methylation (Fig. 5c) and miRNA expression (Fig. 5d). This combined analysis highlighted Sorbs1 as a potential target of epigenetic regulation, with 31 significantly altered CpG sites mapped to the gene. Sorbs1 expression has been previously linked to body mass index, with reduced expression associated with increased adiposity [44]; our findings further suggest that DNA methylation may contribute to its transcriptional regulation. Although no miRNA-mediated regulation was detected for *Sorbs1*, other key genes such as *Insr* showed evidence of coordinated regulation by both DNA methylation and miRNAs, consistent with earlier reports [45,46]. Taken together, these multi-layered visualizations provided by *metaKEGG* facilitate an integrated interpretation of gene expression, epigenetic regulation, and pathway dynamics, thereby supporting mechanistic insights into the differential metabolic phenotypes of DR and DP NZO mice.

### 2.9 Comparing metaKEGG to existing solutions

A number of tools have been developed to extend and enhance the utility of KEGG pathway analysis, each adopting different strategies for mapping identifiers, reconstructing pathway structure, and visualizing omics results. KEGG Mapper remains one of the most widely used options, provided directly by the KEGG team as a suite of mapping utilities that allow users to highlight gene or compound identifiers within the native KEGG pathway viewer. Although straightforward, KEGG Mapper offers primarily static overlays and does not support simultaneous visualization of multiple omics modalities.

Several external tools have attempted to provide greater flexibility. One of the earliest was KEGGtranslator [47], a standalone Java application which enables pathway conversion but does not preserve the original KEGG node structure. Tools within the R ecosystem have been particularly influential: KEGGgraph [48] and Graphite [49] provide graph-based representations and topological analysis of KEGG pathways. Graphite, in particular, improves pathway reconstruction across multiple databases and has been shown to outperform earlier approaches in signaling pathway modeling. However, these tools prioritize computational analysis and require substantial R expertise rather than offering intuitive visual exploration.

More recent visualization-focused tools include ggkegg, which reconstructs KEGG diagrams using ggplot2 and ggraph, enabling highly customizable and publication-quality graphics. Its capabilities range from gene highlighting and hierarchical layout adjustments to multi-scale mapping and single-cell data integration. Still, these features require detailed knowledge of the underlying code and extensive configuration, posing a barrier to users without strong R proficiency. KEGGCharter [50] addresses more specialized needs, particularly for KO-based metagenomic workflows. It provides a console-based solution to visualize KO abundance profiles on KEGG pathways. Nevertheless, it remains tightly coupled to KO-centric analysis and is not readily extensible to multi-omics studies requiring concurrent gene-, miRNA-, methylation-, and metabolite-level inputs.

A widely used tool in this domain is Pathview [51], which retrieves KGML files, reconstructs KEGG layouts programmatically, and overlays user-supplied omics values. Although broadly adopted, Pathview does not preserve KEGG's native graphical structure in all cases and visualizes only one omics layer at a time. Beyond the R ecosystem, Cytoscape [52] serves as a powerful and widely used platform for biological network analysis, with plugins such as KEGGscape [53] enabling the import of KEGG pathways as editable graphs. However, Cytoscape's flexibility comes at the cost of substantial setup and a steep learning curve, and the loss of KEGG's original pathway layout.

Across these tools, a common limitation is the lack of dynamic support for multiple data layers and, more importantly a subdivision/splitting of KEGG nodes to accommodate the multiple gene symbols associated with a single node. When node subdivision exists at all, such as in ggkegg [54], KEGGCharter, or specific use cases of KEGG Mapper, it is typically static, limited to predefined patterns or single-condition color scales. The number, order, and interpretation of node subdivisions in existing tools are typically fixed and cannot adapt automatically to user input. In contrast, *metaKEGG* implements data-driven dynamic node splitting, whereby KEGG gene and compound boxes are programmatically subdivided into an arbitrary number of segments, each representing a distinct omics modality or enrichment workflow. The structure, number, and associated metadata of these subdivisions are computed directly from user-provided data, allowing simultaneous visualization of transcriptomic, DNA methylation, miRNA, metabolomic, or other enrichment results within a single node. To our knowledge, no existing KEGG visualization tool offers this degree of flexibility, scalability, and fidelity to the original KEGG layout, distinguishing *metaKEGG* from static overlay approaches. A comparative overview of available solutions is provided in Supplementary Table S2.

## 3 Conclusion

We present *metaKEGG*, a novel and innovative software package designed to advance pathway enrichment analysis by offering extended visualization routines, pipeline integration, metadata analysis, and combination methods. *metaKEGG* addresses a critical limitation in existing tools - the inability to automatically and dynamically access all gene symbols aggregated under individual KEGG nodes across metabolic pathways. Using our innovative solutions, we extended our algorithm to provide a set of pipelines that would allow users to explore their results in greater depth than what is typically available. The fully integrated pipelines ensure robustness and reproducible results, with a variety of output files and logging. To further improve accessibility and usability, we have developed a web app that allows users to access the full functionality of our algorithm in an intuitive graphical interface suitable for users across all levels of computational expertise. With the rise in popularity and applications of artificial intelligence (AI) in research, we believe that *metaKEGG* would benefit from AI implementations, such as chat functionalities and results interpretation.

## Authors’ contributions

ML - Writing-review & editing, Writing-original draft, Visualization, Data curation, Methodology, Software, Investigation, Formal analysis, Conceptualization; NH - Writing-review & editing, Methodology, Data curation, Validation, Investigation; JG - Writing-review & editing, Methodology, Data curation, Validation; MU - Writing-review & editing, Methodology, Data curation, Validation; TB - Software, Validation, Resources, Writing – review and editing; JT - Software, Validation, Resources, Writing – review and editing; AS - Writing-review & editing, Supervision, Methodology, Resources, Funding acquisition, Conceptualization; HV - Writing-review & editing, Supervision, Methodology, Resources, Funding acquisition, Conceptualization, Project Administration.

All authors reviewed and approved the final version of the manuscript.

## Funding

AS received funding from the German Ministry of Education and Research (DZD, Grant: 82DZD00302, 82DZD03D03), and the State of Brandenburg. HV received funding from the German Ministry of Education and Research (DZD, Grant: 82DZD03I04). AS and HV received funding from the European Union’s Horizon Europe Research and Innovation Programme (OBELISK grant agreement 101080465).

## Data and code availability

metaKEGG (https://metakegg.apps.dzd-ev.org) is an open-source project and is available and maintained on GitHub (https://github.com/dife-bioinformatics/metaKEGG) and published under the AGPL-3.0 license. metaKEGG is released via the Python packaging index (PyPI): https://pypi.org/project/metaKEGG. Extensive documentation on metaKEGG is available at https://github.com/dife-bioinformatics/metaKEGG/blob/master/README.md. Example input and output files are available at the project’s repository on GitHub (https://github.com/dife-bioinformatics/metaKEGG/tree/master/examples). metaKEGG can be used through our web interface at (https://metakegg.apps.dzd-ev.org). The metaKEGG web interface (meta-kegg-web-wrapper) is an open-source project and is available and maintained on GitHub (https://github.com/DZD-eV-Diabetes-Research/meta-kegg-web-wrapper) and published under the MIT license.

## Data handling and security

The web-based application of metaKEGG assigns a unique URL upon every visit through a UUID4 identifier. The URL can be used to revisit an analysis or share analysis results with other researchers. These 128-bit random identifiers make unauthorized access through enumeration computationally impossible and without the complete pipeline URL, users cannot access pipeline configurations or results. Uploaded input files cannot be viewed or downloaded, even if the pipeline URL is exposed; only the aggregated results are available. This prevents scenarios where sensitive input data would be extracted by unauthorized parties, hence the pipeline runs are protected. At any point, users have the option to delete the pipeline performed, with all associated result files and settings. Otherwise, pipelines automatically expire and are deleted after they reach the retention-period limit. This threshold is set at 12 hours (or 720 minutes). The platform operates without a per-user authentication implementation, hence is completely anonymous and minimizes data exchange between the user and the platform and offers. More information on data security can be found in the web application’s project repository (https://github.com/DZD-eV-Diabetes-Research/meta-kegg-web-wrapper/blob/main/README.md).

## Benchmarking

We have performed basic benchmarking for the available pipelines for metaKEGG, as to orient the users in the expected duration of their analysis. The timing includes the instantiation of the class and the respective wrapped pipeline. The libraries are already loaded and the parameters set. The timing of the simulated data was performed for each of the available metaKEGG pipelines (Fig. S14a). There is one pathway mapped per pipeline with 130 genes. We repeated the benchmarking for the NZO DP/DR mouse study (Fig. S14b) where we visualized 4 pathways with 176, 83, 65 and 70 genes, respectively. Due to pipeline design, the implemented 0.5s delay per pathway was deducted from the reported timings as to reflect true runtime.

## Quantification and statistical analyses

The pipeline of RNAseq is originally presented in [55], while the mouse methylation, miRNA, and lipidome sample preparation and preprocessing is found in Sehgal et al. [26]. Briefly, sequencing reads were aligned to the reference genome after adapter trimming and were normalized by generating FPKM values from the raw reads. Low-abundance genes were excluded using FPKM-based filtering criteria (FPKM > 1 in at least one group), to minimize technical variability and enhance data robustness. Comparison between the two groups was performed with the Mann-Whitney U test. P values lower than 0.05 were considered significant and were used to classify genes as differentially expressed which were then used for downstream analyses, namely, functional enrichment analysis. DNA methylation (p < 0.05) and miRNA expression (p < 0.05) data were overlapped with the differentially expressed genes via joining of the annotated genes in the manifest file of the MM285 mouse methylation array or via target gene prediction for miRNAs of experimentally validated miRNA-mRNA interaction.

## Declaration of competing interest

The authors declare that they have no known competing financial interests or personal relationships that could have appeared to influence the work reported in this paper.

## Data Availability

Repository links and further information can be found in the "Data and code availability" section. GitHubSource Code (Original data)
