# Supplementary material for: metaKEGG: A comprehensive algorithm package to visualize multi-omics pathway enrichment
Source: Biochem Biophys Rep. 2026 Aug 25;47:102723. doi: 10.1016/j.bbrep.2026.102723 (PMC13543111; doi:10.1016/j.bbrep.2026.102723)
Supplement: Supplementary file 1 [file mmc1.docx]

Supplementary Information for

**metaKEGG: A comprehensive algorithm package to visualize multi-omics pathway enrichment**

Michail Lazaratos^1,2,*^, Neele Haacke^1,2^, Jasmin Gaugel^1,2^, Miriam Ulz^1,2^, Tim Bleimehl^1^, Justus Täger^1^, Annette Schürmann^1,2,3^ & Heike Vogel^1,2^

**Institutions:**

1) German Center for Diabetes Research (DZD e.V.), München-Neuherberg, Germany

2) Research Group Nutrigenomics of Obesity and Department of Experimental Diabetology, German Institute of Human Nutrition Potsdam-Rehbruecke, Nuthetal, Germany

3) Institute of Nutritional Sciences, University of Potsdam, Nuthetal, Germany

***Corresponding author:**

Michail Lazaratos

Arthur-Scheunert-Allee 114-116

14558 Nuthetal, Deutschland

E-mail: Michail.Lazaratos@dife.de

Tel: +49 33200 88 2533
Supporting Information contains:

- Supplementary Methods

- Supplementary Tables S1 – S2

- Supplementary Figures S1 – S14

**Supplementary Methods**

**Input files**

Starting with the pathway enrichment analysis results, a user would require to provide a Microsoft © Excel file with two sheets (Fig. S2). Sheet (1) (Fig. S2a) contains the enrichment results (default name *pathways*), and sheet (2) (Fig. S2b) contains the gene or transcript (Fig. S2c) information (default name *gene_metrics* or *transcript_metrics*). For the development of this tool we applied DAVID through the DAVID-WS web service [1, 2] using official gene symbols. The gene/transcript information sheet contains a minimum of two columns, the official gene symbol and the mappable metric, which most of the time is the log_2_FC, and an optional ENSEMBL gene or transcript ID column. In the case of metadata files, *metaKEGG* can map DNA methylation or miRNA information on the KEGG pathways. Metadata files can be provided as Microsoft © Excel, tab-separated values (TSV) or comma-separated values (CSV) files, and contain a CG/miRNA ID column (Fig. S2d, e), the associated gene symbol column, a p-value column with a log_2_FC being optional.

Sample files are available in the GitHub repository of the project (<https://github.com/dife-bioinformatics/metaKEGG/tree/master/examples>)

**Metadata – Probe correction setting**

To provide a more universal solution to methylation arrays, we have implemented a feature in the form of an input parameter called “probes_to_cgs” which will split the CG probes names by the underscore (“_”) character and keep the first part, essentially correcting for different probe chemistry that could occur in the same position. Example format is cg00000000_BC21 and cg00000000_TC21, which would be counted as two separate probes targeting the same gene. Using the argument “probes_to_cgs”, the probes become cg00000000 & cg00000000, and duplicated entries per gene are eliminated, essentially counting one probe for the target gene.

Table S1. Metadata types and profile legends for their corresponding metaKEGG analysis.

| Metadata annotations | | |
| --- | --- | --- |
| Differentially methylated genes | miRNA target genes | Differentially methylated + miRNA target genes |
| DEG with DMP | DEmiR target gene | DEG with DMP and DEmiR target gene |
| DEG without DMP | No DEmiR target gene | DEG without DMP and DEmiR target gene |
|  |  | DEG with DMP and no DEmiR target gene |
|  |  | DEG without DMP and no DEmiR target gene |

Table S2. Comparative table summarizing features metaKEGG and already existing KEGG visualization libraries/programs. The capability of each software to process multi-omics, node-splitting, KEGG layout preservation, way of operation or deployment and automation are being compared. The term “Automated” indicates software capable of autonomous operation following parameter definition, without the need for additional coding, preparation steps, or setup procedures.

| **Tool** | **Multi-omics Visualization** | **Node Splitting** | **Dynamic / Data-Driven Node Splitting** | **Preserves Native KEGG Layout** | **Operation /**  **Deployment** | **Automated** | **Notes** |
| --- | --- | --- | --- | --- | --- | --- | --- |
| **KEGG Mapper [1]** | Multiple layers manually configured | Static (input per node) | No | Yes | GUI | No | KEGG powered / integrated |
| **KEGGtranslator [2]** | No | No | No | Partially  (reconstructed graph) | GUI | No | Pathway structure not preserved fully |
| **KEGGgraph [3]** | No | No | No | No (graph abstraction only) | R | No | Topology oriented; microarray data mapping |
| **Graphite [4]** | No | No | No | No (reconstructed graph) | R | No | Topology oriented; little visual capability |
| **Pathview [5]** | Multiple layers manually configured | Static (single color or gradient per node) | No | Yes/Partial reconstruction | R | No | Complex usage; static mapping; requires substantial coding |
| **ggkegg [6]** | Multiple layers manually configured | Static (single color or gradient per node) | No | Yes/Partial reconstruction | R | No | Complex usage; feature-rich; requires substantial coding; scRNA functionality |
| **KEGGCharter [7]** | Mostly metagenomics | Yes (static, KO-centric) | No | Yes | R | Yes | Metagenomics oriented; not general-purpose for multi-omics |
| **Cytoscape + KEGGscape [8, 9]** | One layer at a time | No | No | No/Network reconstruction | GUI | No | Complex usage; Pathway parser; Edge features; Flexible but not automated; Graphical interface; Pathway merging |
| **metaKEGG** | **Yes (automated visualization across pipelines)** | **Yes (dynamic)** | **Yes** | **Yes** | Python/Web interface | Yes | Dynamic node partitioning; transcript mapping;multiomics layers; automated |

Figure S1. Algorithm flow schemes for metaKEGG’s pipelines. (a) Gene expression (b) Transcript expression (c) Bulk RNAseq mapping (d) Multiple inputs (e) Methylated genes (f) DMPs per gene (g) miRNA target genes (h) DEmiRs per gene (i) Methylated + miRNA target genes.

Figure S2. metaKEGG’s input files format. For simplicity, we show streamlined and generalized example formats with arbitrary gene IDs, transcript IDs, cg IDs and miRNA IDs. (a) Example format for the Excel sheet containing the pathway information. This sheet should contain the results of a DAVID pathway enrichment analysis, or be adapted to have a matching format as depicted. (b) Example Excel sheet containing the DEgenes used for the pathway enrichment analysis. (c) Example Excel sheet containing the DEtranscripts used for the pathway enrichment analysis. (compatible only with analysis “Transcript expression”). (d) Example file containing the methylation metadata used for analysis “Methylated genes”,” DMPs per gene”, and “Methylated + miRNA target genes”. The file format for the metadata can one of .csv, .tsv, or .xlsx (e) Example file containing the miRNA metadata used for analysis “miRNA target genes”,” DEmiRs per gene”, and “Methylated + miRNA target genes”. The file format for the metadata can one of .csv, .tsv, or .xlsx (f) Example Excel sheet for the bulk mapping using RNAseq data as the gene information and the list of pathways to be mapped. The list of the pathways must comply with the depicted format.

Figure S3. metaKEGG web app overview. The web app is publicly available under the address (<https://metakegg.apps.dzd-ev.org>) and encapsulates the full capabilities of metaKEGG in 6 distinct steps.

Figure S4. Gene expression. Example visualization of analysis “Gene expression” for randomly generated log_2_FC data for genes belonging in the Non-alcoholic fatty liver disease pathway from Mus Musculus (KEGG ID: mmu04932).

Figure S5. Transcript expression. Example visualization of analysis “Transcript expression” for randomly generated log_2_FC data for genes belonging in the Non-alcoholic fatty liver disease pathway from Mus Musculus (KEGG ID: mmu04932).

Figure S6. Bulk RNAseq mapping. Example visualization of analysis “Bulk RNAseq mapping“ for randomly generated log_2_FC data for genes belonging in the Non-alcoholic fatty liver disease pathway from Mus Musculus (KEGG ID: mmu04932). The difference between analysis types “Bulk RNAseq mapping“ and “Gene expression” is that bulk mapping does not require an enrichment file. It can be used directly with the RNAseq data, and a list of pathways a user wishes to visualize.

Figure S7. Gene expression with compounds. The term “compounds” is used here to describe the metabolites, lipids and small molecules that KEGG organizes in its “KEGG COMPOUND” term, and is described in the Methods section of the main text. Example visualization of analysis “Gene expression” for randomly generated log_2_FC data and additional compounds detected for genes belonging in the Non-alcoholic fatty liver disease pathway from Mus Musculus (KEGG ID: mmu04932).

Figure S8. Analysis of multiple inputs. Example visualization of analysis “Multiple inputs” for randomly generated log_2_FC data for genes belonging in the Non-alcoholic fatty liver disease pathway from Mus Musculus (KEGG ID: mmu04932). Three input files were used, resulting in 7 possible states for a given gene to be in.

Figure S9. Differentially methylated genes. Example visualization of analysis “Differentially methylated genes“ for randomly generated log_2_FC data, and methylation annotations for genes belonging in the Non-alcoholic fatty liver disease pathway from Mus Musculus (KEGG ID: mmu04932). The two possible states for static methylation mapping are “DEG with DMP” for a gene targeted by at least one probe, and “DEG without DMP” for a gene without any corresponding probes.

Figure S10. DMPs per gene. Example visualization of analysis “DMPs per gene” for randomly generated log_2_FC data and methylation annotations for genes belonging in the Non-alcoholic fatty liver disease pathway from Mus Musculus (KEGG ID: mmu04932). The number of probes (or DMPs) per gene are grouped in preselected bins, adjusted for the maximum number of probes (or DMPs) and are assigned a unique color code. (b) Histogram of the grouped DMPs per gene. (c) Histogram of the absolute count of DMPs per gene.

Figure S11. miRNA target genes. Example visualization of analysis “miRNA target genes” for randomly generated log_2_FC data for genes and miRNA annotations belonging in the Non-alcoholic fatty liver disease pathway from Mus Musculus (KEGG ID: mmu04932). The two possible states for static miRNA mapping are “DEmiR target gene” for a gene targeted by at least miRNA, and “No DEmiR target gene” for a gene without any corresponding miRNAs.

Figure S12. DEmiRs per gene. Example visualization of analysis “DEmiRs per gene” for randomly generated log_2_FC data and miRNA annotations for genes belonging in the Non-alcoholic fatty liver disease pathway from Mus Musculus (KEGG ID: mmu04932). The number of differentially expressed miRNAs (DEmiRs) per gene are grouped in preselected bins, adjusted for the maximum number of DEmiRs and are assigned a unique color code. (b) Histogram of the grouped DEmiRs per gene. (c) Histogram of the absolute count of DEmiRs per gene.

Figure S13. Methylated + miRNA target genes. Example visualization of analysis “Differentially methylated + miRNA target genes” for randomly generated log_2_FC data and methylation & miRNA annotations for genes belonging in the Non-alcoholic fatty liver disease pathway from Mus Musculus (KEGG ID: mmu04932). The four possible annotations are “DEG with DMP and DEmiR target gene”, “DEG without DMP and DEmiR target gene”, “DEG with DMP and no DEmiR target gene”, and “DEG without DMP and no DEmiR target gene”.

Figure S14. metaKEGG benchmarking. (a) Single pipeline benchmarking including the class instantiation and pipeline run for the example files found in the project’s repository. The default pathway file features one pathway with 130 genes. (b) Timing of the analysis performed on the NZO DP/DR mouse model. The enrichment analysis benchmarked here features 4 pathways with 176, 83, 65 and 70 genes respectively. A time delay of 0.5s per pathway has been subtracted from the final timing as to reflect the true runtime.

**References**

1.	Jiao, X., et al., *DAVID-WS: a stateful web service to facilitate gene/protein list analysis.* Bioinformatics, 2012. **28**(13): p. 1805-1806.

2.	Fresno, C. and E.A. Fernández, *RDAVIDWebService: a versatile R interface to DAVID.* Bioinformatics, 2013. **29**(21): p. 2810-2811.
